# Rectal ulcer in a hemodialysis patient receiving Kayexalate^®^

**DOI:** 10.1002/ccr3.4043

**Published:** 2021-03-13

**Authors:** Julia Collot, Tayeb Salaouatchi, Fabienne Rickaert, Albino Floriani, Benoit Buysschaert, Maria Mesquita, Eric Godon

**Affiliations:** ^1^ Nephrology and Dialysis Clinic Centre Hospitalier Regional de Huy Huy Belgium; ^2^ Nephrology and Dialysis Clinic Internal Medicine Department Centre Hospitalier Universitaire CHU‐Brugmann Brussels Belgium; ^3^ Department of Pathology Centre Hospitalier Universitaire Liège Belgium; ^4^ Department of Gastroenterology Centre Hospitalier Regional de Huy Huy Belgium; ^5^ Centre Hospitalier Regional de Huy Huy Belgium

**Keywords:** hemodialysis, hyperkalemia, kayexalate crystals, kayexalate^®^, rectal ulcer

## Abstract

Kayexalate can cause severe unrecognized GI lesions. Diagnosis of kayexalate crystals in GI biopsy samples is important. Pathologists and clinicians should work hand in hand. New drugs should be available to all patients to treat hyperkalemia.

## Graphical abstract

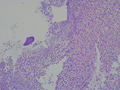

## 1 INTRODUCTION

Kayexalate^®^ is associated with gastrointestinal injury that can be fatal. Physicians must be aware of the adverse events when prescribing this therapy. Clinical data and medication history should follow the biopsy material given to the pathologist, who will look for Kayexalate^®^ crystals. Pathologists and physicians should work hand in hand.

Hyperkalemia may cause fatal arrhythmia in patients undergoing maintenance hemodialysis (HD).^1^ In the majority of patients receiving HD, it is possible to avoid death from hyperkalemia with dietary therapy and adequate HD. However, some patients with oliguria or anuria receiving HD require treatment with an ion‐exchange resin such as Kayexalate^®^ to prevent hyperkalemia.

Potassium plasmatic concentration is influenced by many other factors than renal insufficiency such as nonsteroidal anti‐inflammatory drugs (NSAIDs), angiotensin‐converting enzyme (ACE) inhibitors and angiotensin II receptor blockers (ARBs), glycemia, acidosis, hemolysis, and adrenal insufficiency.

The use of Kayexalate^®^ in the treatment of hyperkalemia may be associated with serious gastrointestinal (GI) adverse events. Side effects, with sometimes lethal sequelae, have been shown to occur in both the upper and lower GI tract in association with Kayexalate^®^ use, and range from mild GI bleeding to perforation.^2^

We report a case of multiple GI bleeding in a HD patient treated with Kayexalate^®^ for chronic hyperkalemia.

## 2 CASE REPORT

A 69‐year‐old woman on maintenance hemodialysis (HD) with a history of type 2 diabetes mellitus, hypertension, ischemic cardiomyopathy, and chronic obstructive pulmonary disease (COPD) was treated by Kayexalate^®^ for a refractory hyperkalemia which was attributed to chronic abuse of NSAIDs and noncompliance to the prescribed diet.

The patient was also known for a history of two episodes of upper GI bleedings. First, on a large hemorrhagic duodenal ulcer and second one on a gastric ulcer, both linked to NSAID consumption and treated successfully with proton pump inhibitor (PPI).

**FIGURE 1:**
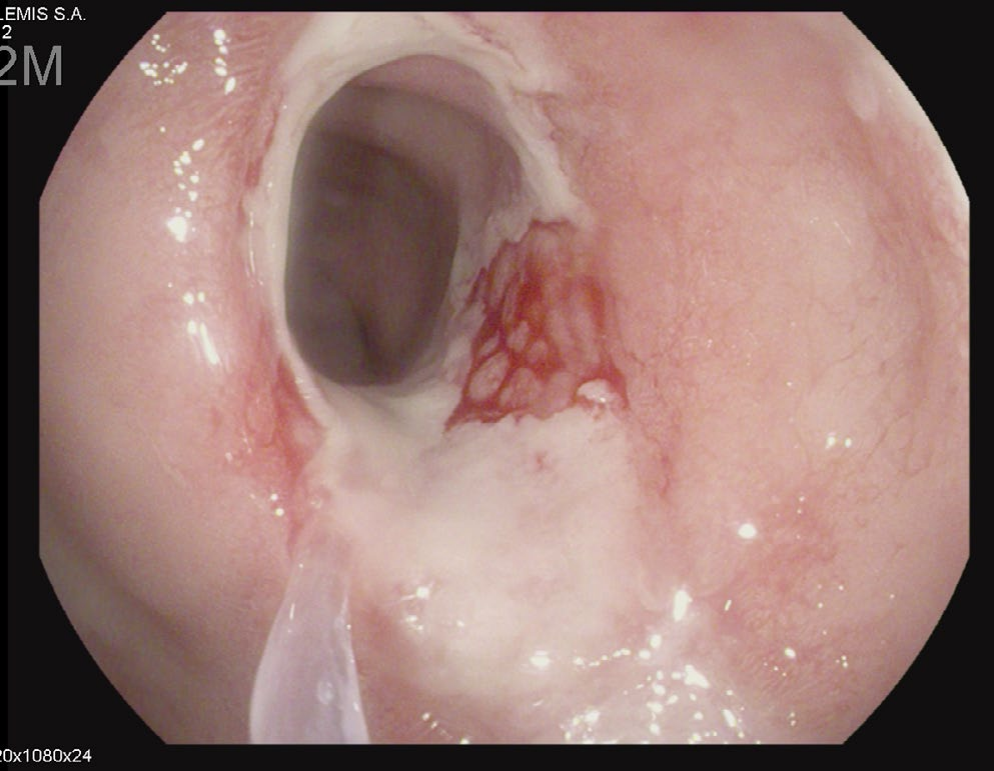
Circumferential ulcer in the lower third of the rectum

The patient presented a third episode of GI bleeding with hematochezia. Laboratory tests showed: severe chronic renal failure, glomerular filtration rate at 10ml/min/1.73m^2^, urea 190 mg/d (normal values (NV) 13‐47), creatinemia 3.9 mg/d ( NV 0.70‐1.20), no acidosis, phosphate levels 1.87 mmol/L(NV 0.75‐1.39), kaliemia 6 mmol/L (VN 3.5‐4.5), and Hb 8.5g/dL (NV 12‐16 g/dL). The rectoscopy revealed a circumferential ulcer of the inferior rectum (Figure 1).

**FIGURE 2:**
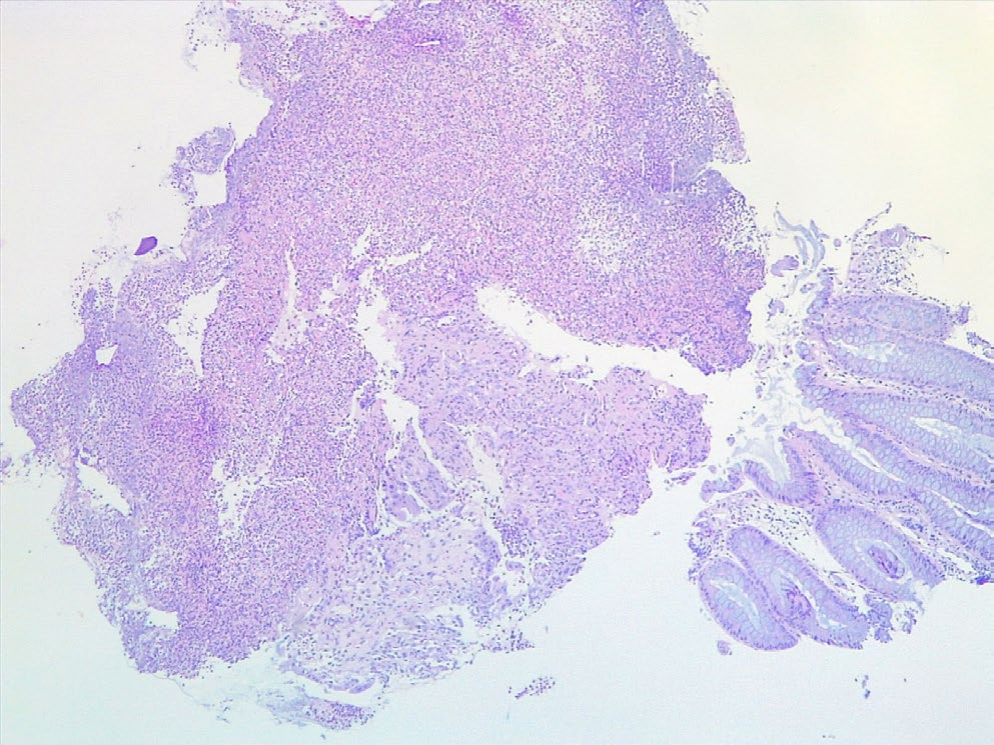
Refractile basophilic crystal with typical fish scale morphology

**FIGURE 3:**
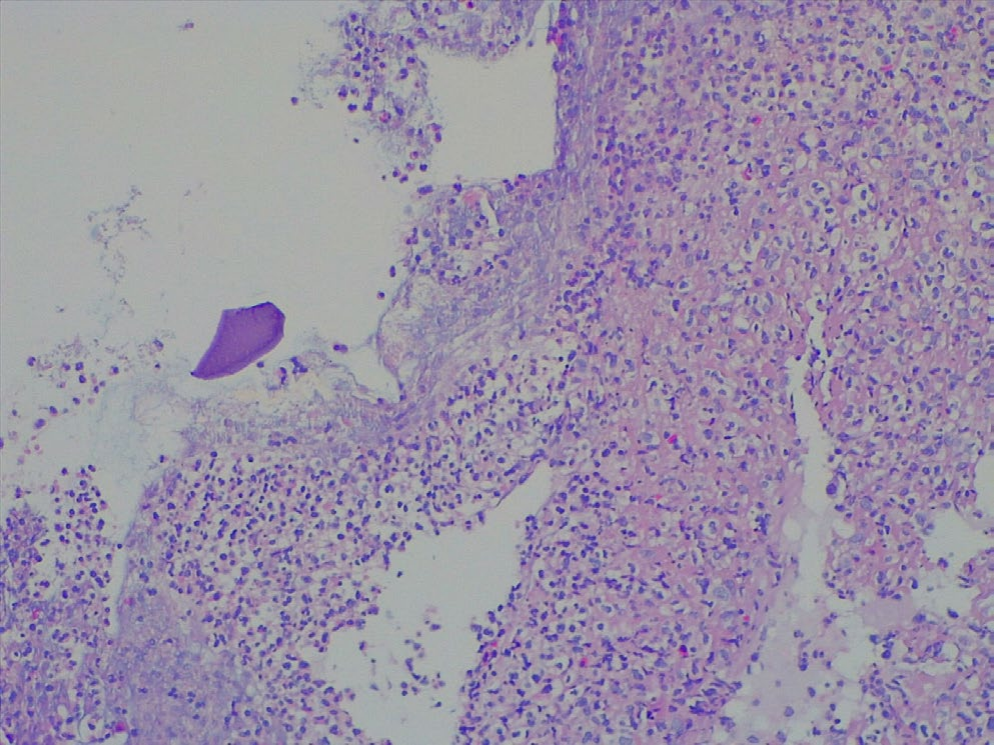
Colon mucosal ulceration with a crystal in the exudate

Histological examination of the rectal biopsies showed rectal mucosal ulcerations and rectangular basophilic crystals like “fish scales” adherent to the surface epithelium (Figure 2) and colon mucosal ulceration with a crystal in the exudate (Figure 3). No vascular disease was seen on the biopsies.

So, the diagnosis of Kayexalate^®^‐induced rectal injury was made on the basis of microscopic features, her clinical history, and medication records. Kayexalate^®^ doses were first reduced; however, it had to be continued for some time because of noncontrolled hyperkalemia.

For the time being, the patient is on HD thrice a week, she does not take any more NSAIDs, and she pays attention to her diet and does not require anymore a potassium binder. Since 6 months, she has no GI complaints and no digestive bleeding was observed.

## 3 DISCUSSION

The GI tract is the target of several types of medical drugs including NSAIDs, iron pills, paclitaxel (Taxol^®^), mycophenolate mofetil (Cellcept^®^), bisphosphonates, colchicine, and oral resins such as Kayexalate^®^.^3^

Kayexalate^®^ is a potassium‐binding resin commonly used to treat mild acute or chronic hyperkalemia by increasing the excretion of potassium in stool. It can be administrated orally or locally as an enema, and it is sometimes associated with sorbitol to prevent fecal impaction. It is a common drug prescribed in ESRD and hemodialysis patients.^4^

Chronic kidney disease, end‐stage renal disease, solid organ transplant, and postoperative status are risk factors for GI injury associated with kayexalate use.^5^

Hence, our patient was at high risk to develop Kayexalate^®^‐related GI complications because she had ESRD. Nevertheless, this drug was prescribed because of uncontrolled hyperkalemia and the risk of fatal cardiac arrhythmias. Our patient had persistent hyperkalemia (blood levels > 6 mmol /L) because of NSAID automedication in the setting of ESRD and noncompliance to the diet prescribed, and kayexalate was prescribed to maintain the potassium blood levels <6 mmol/L.

The common side effects of Kayexalate^®^ are loss of appetite, upset stomach, nausea, vomiting, constipation, or diarrhea.^6^ Despite the tolerability and efficacy of Kayexalate^®^ for long‐term management of hyperkalemia in patients with chronic kidney disease,^7^ some authors doubt about the effectiveness and safety of this drug.^8^, ^9^, ^10^

In addition to the common side effects of Kayexalate^®^, mucosal ulceration including wall perforation and postinflammatory stricture formation is among the serious complications which may present as a clinical emergency.^11^, ^12^, ^13^, ^14^, ^15^, ^16^, ^17^ Probably, if diagnosed early, a simple colonic ulcer can be diagnosed in some patients.^18^ Intestinal ischemia and necrosis has also been reported.^19^ Rectal ulcers and rectal stenosis can be caused by foreign body reaction to Kayexalate^®^ crystals.^20^ ^21^

In fact, review of the literature shows that this resin can induce not only intestinal, colonic, and rectal ulcers, necrosis, and perforations but that the upper gastrointestinal tract can also be injured because of this drug.^11^, ^22^

Our patient had a circumferential ulcer in the lower third of the rectum, a complication related to the use of Kayexalate^®^ as shown at the histopathologic examination of the biopsy.

The incidence of gastrointestinal injuries related to Kayexalate^®^ (with or without sorbitol) varies in different population studies and according to the dose prescribed.

The estimated incidence of intestinal injury when sorbitol is added to Kayexalate^®^ varies between 0.27% and 1.8%.^19^, ^22^ The incidence of gastrointestinal tract injuries because of Kayexalate^®^ in CKD and dialysis patients is not known but seems very low.

A retrospective cohort study based on the Swedish Renal Registry (SRR) showed that Kayexalate^®^ is associated with a dose‐dependent increase in the incidence of serious GI adverse events for CKD patients. In addition, Kayexalate^®^ use was associated with a higher rate of laxative dispensations. These associations were more evident in patients prescribed per label Kayexalate^®^ dosages.^23^

Another study in older adults initiating Kayexalate^®^ showed higher incidence of hospitalizations for serious GI adverse events among Kayexalate^®^ users compared with nonusers.^24^

Kayexalate^®^‐induced intestinal ischemia remains an under‐recognized, easily avoidable complication, associated with significant morbidity and mortality. Physicians who routinely use this agent in sorbitol or without sorbitol should be aware of its life‐threatening complications. As soon as the diagnosis is done, the drug should be stopped in order to avoid further fatal complications. For the moment, in many countries in Europe, alternating drugs are not available and clinicians are obliged to continue prescribing kayexalate. Our patient was treated with Kayexalate^®^ (without administration of sorbitol) and we continued this drug, prescribing lower doses because of noncontrolled hyperkalemia.

The most common medication resins utilized in renal patients are Sevelamer and Kayexalate^®^ that chelate phosphate and potassium, respectively. Both are associated with mucosal injury. Lesions seen in resin‐induced gastrointestinal damage may overlap with those seen in inflammatory bowel disease, ischemic colitis, infectious colitis, and microscopic colitis. The distinctive feature of resin mucosal damage is the identification of resin crystals. However, this was reported by the pathologist roughly in 75% of the cases.^25^

The classic morphology to diagnose these crystals is as follows: Kayexalate is rectangular in shape and purple on hematoxylin‐eosin staining (H&E) and has “fish scales” or internal demarcations that appear similar to external surface of a fish. Sevelamer (Renvela^®^ and Renagel^®^) is also rectangular in shape and usually 2‐toned on H&E, with pink center and yellow edges; it also has fish scales.^25^

Classic morphology is the benchmark for identifying the resins, but color, location, and fish scale pattern can deviate from the norm, making proper identification a challenge. Sometimes, ancillary tools are required to distinguish them. Acid‐fast bacillus special stain is the most reliable ancillary tool; it stains Kayexalate in black and Sevelamer in magenta. Discussion with the clinician about the medication taken by the patient is useful. It is critical for pathologists to be aware of the typical and atypical presentations of medication resins.^25^

The typical histopathologic findings in Kayexalate^®^‐induced necrosis include mucosal ulceration, necrosis, and the presence of polygonal basophilic retractile crystals with a “fish scale” appearance..^5^, ^26^ For pathologists, identifying the kayexalate crystals in necrotic gastrointestinal tissue is important, because early feedback to the clinician can lead to the cease of medication and avoidance of more serious adverse outcomes. Hence, clinicians and pathologist should work hand in hand.

Before she was taken for dialysis treatment because of ESRD, our patient presented antral and duodenal ulcers. Our pathologist took a second look in the gastric and duodenal biopsy samples in search once again for kayexalate^®^ crystals but found none. This could be a false‐negative result linked to biopsy sampling. It was suggested that these upper GI ulcers could be due to NSAID consumption.

Kayexalate^®^ is commonly prescribed for the treatment of hyperkalemia; however, two new potassium‐binding agents, sodium zirconium cyclosilicate and patiromer, appear to have greater binding selectivity for potassium compared with that of SPS, but they are not yet available in all countries and for the treatment of all causes of hyperkalemia.^27^, ^28^, ^29^

In Belgium and in many countries of Europe, sodium zirconium cyclosilicate (ZS‐9) is not available and patiromer is not freely commercialized and can be prescribed only by a nephrologist or cardiologist. All our dialysis patients who have chronic uncontrolled hyperkalemia receive 15 g of kayexalate during their main meal, the days without dialysis.

Since for the moment in many countries we do not have an alternative drug to control hyperkalemia in hemodialysis patients, clinicians should be vigilant when a patient taking kayexalate presents GI bleeding. Sometimes, daily dialysis and more emphasis on diet are required to control hyperkalemia in these patients in order to prevent fatal cardiac arrhythmias.

## 4 CONCLUSIONS

Kayexalate is a fairly commonly used drug for hyperkalemia in Europe but some of the side effects could go unrecognized and are underappreciated by some clinicians.

Given the severity of GI complications, the therapeutic role of kayexalate^®^ needs to be re‐evaluated. Although the risk to an individual patient may not be high, the widespread use of this medication may be exposing a large population to potential risks, especially in high risk patients. As it is often given to fragile patients, it is important to use it cautiously to reduce as much as possible lethal side effects of this drug. Our observation suggests that physicians must be aware of the risk of these adverse events when prescribing kayexalate^®^.

Alternative treatments in the management of hyperkalemia in CKD/ESRD are the new armamentarium of potassium binders: patiromer and sodium zirconium cyclosilicate.

These new drugs are not yet available in all countries. Moreover, randomized controlled studies should show the advantage of these new drugs in the protection of the GI tract in patients.

It is critical for pathologists to be aware of the typical and atypical presentations of medication resins, because early feedback to the clinician can lead to the cease of medication and avoidance of more serious adverse outcomes. Recognition and timely diagnosis can be life‐saving. Discussion between clinicians and the pathologist is extremely important and is underutilized in modern medicine. Hence, clinicians and pathologist should work hand in hand.

## CONFLICT OF INTEREST

None declared.

## AUTHOR CONTRIBUTIONS

CJ and MM: have primarily designed and drafted the paper.ST: reviewed the related articles and also contributed in writing the manuscript. RF: wrote the pathology content and obtained the images. FA, BB, and GE: revised the manuscript. All authors have read and approved the final version of the revised paper.

## PATIENT CONSENT

Informed consent was obtained from the patient for the publication of this case report.

## Data Availability

The data that support the findings of this study are available from the corresponding author upon reasonable request.
